# Treatment Patterns, Health Care Resource Utilization, and Health Care Cost Associated with Atypical Antipsychotics or Guanfacine Extended Release in Children and Adolescents with Attention-Deficit/Hyperactivity Disorder in Quebec, Canada

**DOI:** 10.1089/cap.2019.0097

**Published:** 2019-12-02

**Authors:** Jean Lachaine, Leila Ben Amor, Tamara Pringsheim, James Burns, Judy van Stralen

**Affiliations:** ^1^ Faculty of Pharmacy, University of Montreal, Montreal, Quebec, Canada.; ^2^ Department of Psychiatry, University of Montreal, Montreal, Quebec, Canada.; ^3^ Department of Clinical Neurosciences, University of Calgary, Calgary, Alberta, Canada.; ^4^ Department of Psychiatry, University of Calgary, Calgary, Alberta, Canada.; ^5^ Department of Community Health Sciences, University of Calgary, Calgary, Alberta, Canada.; ^6^ Department of Pediatrics, University of Calgary, Calgary, Alberta, Canada.; ^7^ Shire, a Takeda company, Toronto, Ontario, Canada.; ^8^ Center for Pediatric Excellence, Ottawa, Ontario, Canada.; ^9^ Department of Paediatrics, Queen's University, Ottawa, Ontario, Canada.

**Keywords:** risperidone, second-line ADHD therapy, metabolic syndrome

## Abstract

***Objective:*** To assess treatment patterns, health care resource utilization, and health care costs associated with use of atypical antipsychotics (AAPs) or the nonstimulant guanfacine extended release (GXR) after stimulant therapy for attention-deficit/hyperactivity disorder (ADHD). In Canada, GXR is approved as a monotherapy for children and adolescents with ADHD or as an adjunct to stimulants, and AAPs are commonly used off-label as an adjunct to stimulants.

***Methods:*** Health care claims data (January 1, 2007 to March 31, 2016) from Quebec's provincial health plan were assessed for individuals with ADHD, 6–17 years of age, who received ≥1 stimulant followed by a first AAP or GXR prescription (index medication), without a diagnosis for which AAPs are indicated.

***Results:*** Overall, 1327 individuals were included (AAPs, 1098; GXR, 229). Rates of discontinuation, augmentation, or switching of the index medication did not differ between AAPs and GXR during the first follow-up year. Discontinuation rates were significantly lower with GXR than with AAPs during the second year (22.0% vs. 35.9%; *p* = 0.03). GXR and AAPs resulted in similar increases in total health care cost. In GXR users, the increase in prescription drug cost after 6 months was higher than in AAP users, whereas the increase in overall medical cost was higher with AAPs than GXR, owing to more psychiatric department visits.

***Conclusions:*** In children and adolescents with ADHD who used AAPs or GXR after stimulants, secondary treatment changes were similar with both treatments after 1 year, but discontinuation rates were significantly lower with GXR than with AAPs in the second year. The greater increase in prescription cost with GXR was balanced by a greater increase in overall medical costs with AAPs, resulting in no overall difference in total health care cost between the two treatments.

## Introduction

Attention-deficit/hyperactivity disorder (ADHD) is a common pediatric disorder associated with a considerable economic burden. In Canada, the estimated prevalence of ADHD in school-aged children is 4.1%–5.0% (Brault and Lacourse 2012; Centre for ADHD Awareness Canada [CADDAC] 2011), with an estimated cost (based on USA data) to the Canadian economy of >2 billion C$ per year (CADDAC 2011). Guidelines recommend stimulants as first-line pharmacotherapy for ADHD (Canadian Attention Deficit Hyperactivity Disorder Resource Alliance [CADDRA] 2011); however, a considerable proportion of patients receiving stimulants change their regimen, owing to an inadequate response, dose-limiting side effects, comorbid disorders, or personal preferences (Arnold 2000; Stockl et al. 2003; Elvanse Prescribing Information 2014; Equasym Prescribing Information 2014).

Claims database studies from Canada and the USA have demonstrated that, among stimulant-treated children and adolescents with ADHD, the 1-year prevalence of medication switching or augmentation is 19%–23% (Ben Amor et al. 2014; Betts et al. 2014). Other approved therapies used in patients with ADHD include nonstimulants such as guanfacine extended release (GXR) and atomoxetine (ATX). Although not approved for the treatment of ADHD, clonidine and atypical antipsychotics (AAPs) are commonly used off-label in ADHD, especially when stimulants fail.

ADHD is the most common diagnosis associated with an AAP prescription in young people in the USA and Canada, despite not being indicated for this condition (Pathak et al. 2010; Pringsheim et al. 2011b; Sohn et al. 2016a). Furthermore, AAPs were identified as the most common psychotropic medication used to augment a stimulant in a study using medical claims data from children and adolescents with ADHD enrolled in Quebec's provincial health plan (Régie de l'assurance maladie du Québec [RAMQ]) between 2007 and 2012 (Ben Amor et al. 2014). The same study also found AAPs to be the second most common medication when switching from a stimulant (Ben Amor et al. 2014).

Although commonly prescribed in individuals with ADHD, AAPs do not address the core symptoms of the disorder (Tramontina et al. 2009; Zeni et al. 2009), but are generally used to treat comorbid oppositional and aggressive behavior (Aman et al. 2004; Armenteros et al. 2007; CADDRA 2011; Pringsheim et al. 2015). Furthermore, AAPs are associated with hormonal, extrapyramidal, and metabolic side effects, such as weight gain and increase in body mass index (BMI), which can be exacerbated during long-term treatment and result in treatment discontinuation (Ho et al. 2011; Pringsheim et al. 2011a, 2011c; Ben Amor 2012; Rasimas and Liebelt 2012).

The use of AAPs in patients with ADHD is also associated with frequent treatment changes, increased health care resource utilization (HCRU), and higher health care cost (Sikirica et al. 2012b; Lachaine et al. 2014). For example, in the Canadian RAMQ study from 2007 to 2012, all-cause total health care cost increased by 46.6%, 6 months after initiating an AAP compared with 6 months before (Lachaine et al. 2014). Likewise, U.S. data showed that children with ADHD who changed their stimulant regimen to include an AAP incurred mean annual total health care costs of U.S.$6934 compared with U.S.$4748 for those using a non-AAP medication (Sikirica et al. 2012b).

GXR is a nonstimulant therapy approved in Canada and the USA for treatment of ADHD in children and adolescents (6–17 years) as a monotherapy or an adjunct to stimulants (Intuniv prescribing information 2016; Intuniv XR 2016). In Canada, GXR was approved in November 2013 and is the only ADHD medication approved to date in Canada for adjunctive use with stimulants in youth (CADDRA 2011; Intuniv XR 2016).

Clinical trials have established the safety and efficacy of GXR as a monotherapy in patients who are stimulant naive or had prior methylphenidate (MPH) treatment (Huss et al. 2016), and as combination therapy with stimulants after a partial response to prior stimulant monotherapy (Wilens et al. 2012). In children with ADHD and comorbid oppositional defiant disorder, GXR monotherapy improved ADHD core symptoms and oppositional symptoms (Connor et al. 2010). Oppositional symptoms were also reduced in patients with ADHD receiving GXR adjunctively to stimulants (Findling et al. 2014). GXR adjunctive to stimulants has been shown to be a cost-effective option compared with stimulants alone in children with a suboptimal response to stimulant monotherapy in Canada and the USA (Sikirica et al. 2012a; Lachaine et al. 2016).

The objective of this study, a follow-up to the antecedent Canadian RAMQ study conducted from 2007 to 2012 (Lachaine et al. 2014), was to assess treatment patterns and health care costs in children and adolescents (6–17 years of age) with ADHD using an AAP or GXR to augment, or switch from, stimulant therapy.

## Patients and Methods

### Data and patient selection

This retrospective study extracted anonymized claims data for medical services and drug prescriptions from Quebec's RAMQ database. Medical services claims data were derived from the universal health care program (covering the entire Quebec population; ∼8.0 million people in 2014) (Régie de l'assurance maladie du Québec 2014d); prescription claims data were derived from the public prescription drug plan (covering individuals with last-resort financial assistance [i.e. beneficiaries of the social assistance program], individuals without access to a private medication insurance plan at their workplace [∼3.5 million people in 2014], and all individuals ≥65 years of age) (Régie de l'assurance maladie du Québec 2014a, 2014b, 2014c).

Participants were required to have a diagnosis of ADHD, and (i) have received ≥1 stimulant medication between January 1, 2007, and March 31, 2016; (ii) have filled a first prescription for an AAP or GXR (defined as the index treatment) after a filled stimulant prescription (defined as the index stimulant); (iii) have ≥30 days' supply of the stimulant before initiating the index treatment; (iv) be covered by the RAMQ prescription drug plan for ≥6 months before and ≥12 months after initiation of the index treatment; (v) be 6–17 years of age at initiation of the index treatment (defined as the index date); and (vi) meet protocol-defined criteria for augmentation with or switching to the index treatment.

Discontinuation was defined as a gap of ≥30 consecutive days between the end of supply and either the beginning of the following prescription fill or the end of the follow-up period (12 or 24 months after start of the original treatment), whichever occurred earliest. Discontinuation date was defined as the last day of supply before the gap. Augmentation was defined as the event in which a new medication was initiated and was used concomitantly with the original medication for ≥30 consecutive days during the follow-up period. Switching was defined as a prescription fill of a new medication that had an overlap in supply of <30 days with the original medication, or a gap of <30 days between the end of supply of the original medication and the initiation of the new medication.

Patients were excluded if they had a documented psychiatric diagnosis for which AAPs are indicated (including bipolar disorder, mania, schizophrenia, and other psychotic disorders) during the baseline period or follow-up.

### Analyses

**FIG. 1.**
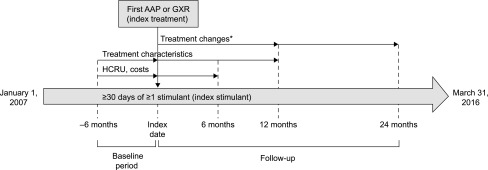
Study design and assessments. *Discontinuation, augmentation, and switching were assessed during the first year of follow-up; discontinuation was also assessed during the second year of follow-up. AAP, atypical antipsychotic; GXR, guanfacine extended release; HCRU, health care resource utilization.

Treatment characteristics were analyzed during the 6-month period before the index date (defined as the baseline period) and the 12-month follow-up (Fig. 1). Treatment patterns of the index treatment (rates of secondary augmentation, secondary switching, and discontinuation) were analyzed during the first year of follow-up. Discontinuation, which can be more frequent during long-term use of AAPs owing to chronic side effects (Rasimas and Liebelt 2012), was also analyzed during the second year of follow-up. All-cause HCRU and health care cost were assessed during the 6-month periods before and after the index date. A subanalysis, including patients with an index date December 1, 2013 (corresponding to the earliest index date in the GXR group), or later was also performed to assess treatment changes, HCRU, and health care cost in the AAP and GXR groups over the same time period.

The overall trend in all-cause health care cost between February 2012 and February 2016, corresponding to the 2-year periods before and after introduction of GXR in the Canadian formulary (February 2014), was evaluated based on all patients who (i) had ≥1 diagnosis of ADHD and ≥1 prescription of a stimulant (with ≥30 days' supply) between February 1, 2012, and February 1, 2016; (ii) were covered by the RAMQ drug insurance plan between February 1, 2013, and February 1, 2015; (iii) were 6–17 years of age at first diagnosis or first stimulant prescription; and (iv) had no documented psychiatric diagnosis in this period for which AAPs are indicated.

### Statistical analysis

Time from initiation of the index treatment to discontinuation, or secondary augmentation or switching during the follow-up period was estimated using Kaplan–Meier analyses, and compared between the AAP and GXR groups using log-rank test. Patients were censored at discontinuation of the initial index treatment when assessing switching, and at discontinuation or switching of the initial index treatment when assessing augmentation. In each treatment group, mean HCRU and health care cost per patient were compared between the 6-month periods before and after the index date using Wilcoxon signed-rank test (0.05 significance level; two-sided). Factors associated with a significant change in cost in the 6 months after the index date were assessed using linear regression, including type of index treatment (AAP or GXR), prescribing physician's specialty, and patient age, sex, last-resort financial assistance status, and number of comorbidities as covariates.

## Results

### Patient disposition and treatment characteristics before initiation of the index treatment

**FIG. 2.**
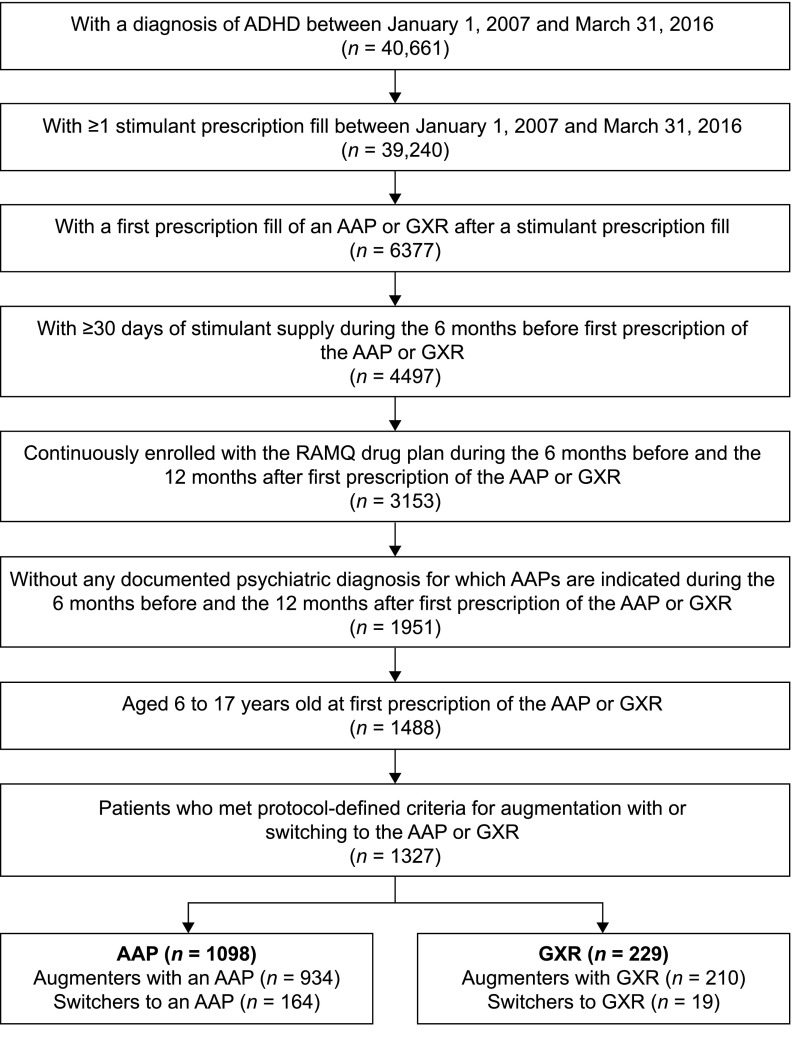
Flow chart of patient selection. AAP, atypical antipsychotic; ADHD, attention-deficit/hyperactivity disorder; GXR, guanfacine extended release; RAMQ, Régie de l'assurance maladie du Québec.

Overall, 6377 patients with ADHD who, between January 1, 2007, and March 31, 2016, had received a first prescription for an AAP or GXR following a stimulant were identified in the RAMQ database (Fig. 2). After applying additional inclusion criteria, the study included 1327 children and adolescents who had augmented their stimulant treatment with an AAP or GXR (*n* = 1144), or switched from their stimulant to an AAP or GXR (*n* = 183). Use of an AAP as the index treatment was more common (*n* = 1098) than use of GXR (*n* = 229).

At index date, mean age in both treatment groups was ∼10 years, most participants were male, and about 50% of participants in each group had at least one comorbidity (Table 1). The AAP group included more recipients of last-resort financial assistance (42.2%) than the GXR group (25.8%). The type of stimulant most frequently used before the index treatment was MPH (generic instant release MPH or extended release MPH [Concerta]) in the AAP group, and extended release (ER) amphetamine (lisdexamfetamine, Vyvanse) in the GXR group (Supplementary Table S1). In both groups, stimulant therapy during the baseline period was mostly prescribed by pediatricians (Supplementary Table S2).

**Table 1.** Demographics and Patient Characteristics

	*AAP (*n* = 1098)*	*GXR (*n* = 229)*
Sociodemographics		
Age, years, mean (SD)^a^	10.7 (3.0)	10.1 (2.2)
Age groups, years, *n* (%)^a^		
6–12	792 (72.1)	185 (80.8)
13–17	306 (27.9)	44 (19.2)
Male, *n* (%)^a^	847 (77.1)	175 (76.4)
Recipients of last-resort financial assistance, *n* (%)^bc^	463 (42.2)	59 (25.8)
Number of comorbidities, *n* (%)^d^		
0	561 (51.1)	124 (54.1)
1	369 (33.6)	79 (34.5)
2	125 (11.4)	20 (8.7)
≥3	43 (3.9)	6 (2.6)
Comorbidity profile, *n* (%)^d^		
Adjustment disorder	97 (8.8)	9 (3.9)
Anxiety disorder	57 (5.2)	9 (3.9)
Conduct disorder	42 (3.8)	12 (5.2)
Depression	42 (3.8)	2 (0.9)
Insomnia	7 (0.6)	4 (1.7)
Learning disability	45 (4.1)	11 (4.8)
Obsessive–compulsive disorder	5 (0.5)	1 (0.4)
Oppositional defiant disorder	19 (1.7)	0 (0)
Pervasive developmental disorders	16 (1.5)	4 (1.7)
Tics	38 (3.5)	10 (4.4)
Substance abuse	17 (1.5)	2 (0.9)
Epilepsy	8 (0.7)	1 (0.4)
Other neurological disorders	7 (0.6)	3 (1.3)
Accidents and injuries	327 (29.8)	66 (28.8)
Asthma	33 (3.0)	3 (1.3)

^a^ At index date.

^b^ Beneficiaries of the social assistance program.

^c^ At the time of study inclusion (first ADHD diagnosis or first stimulant prescription between January 2007 and March 2016).

^d^ During baseline period (6-month period before index date).

AAP, atypical antipsychotic; ADHD, attention-deficit/hyperactivity disorder; GXR, guanfacine extended release; SD, standard deviation.

### Treatment characteristics and changes after initiation of the index treatment

Among patients who used AAPs as the index treatment, 62.3% received risperidone. Among the physicians prescribing AAPs as the index treatment, 31.5% were psychiatrists and 24.6% were pediatricians; in contrast, 50.7% of the GXR prescriptions were issued by pediatricians (Supplementary Table S2). Within 1 year after the index date, the majority of participants in both groups discontinued, augmented, or switched the index treatment (AAPs, 70.0%; GXR, 66.4%), with discontinuation being the most common type of treatment change (AAP, 54.3%; GXR, 55.5%) (Table 2). In both treatment groups, stimulants were the most common medication used for secondary augmentation, whereas secondary switches involved mostly AAPs or stimulants (Table 2). Use of GXR for a secondary treatment change in the AAP group was uncommon (<1%).

**Table 2.** Changes of Index Treatment During 12-Month Follow-Up

*Type of change,* n *(%)*	*AAP (*n* = 1098)*	*GXR (*n* = 229)*
Any change in index treatment^a^	769 (70.0)	152 (66.4)
Index treatment discontinuation	596 (54.3)	127 (55.5)
Index treatment augmentation	247 (22.5)	42 (18.3)
With an AAP	19 (1.7)	8 (3.5)
With GXR	3 (0.3)	—
With a nonstimulant other than GXR	48 (4.4)	2 (0.9)
With a stimulant	177 (16.1)	32 (14.0)
Index treatment switching	135 (12.3)	37 (16.2)
To an AAP	55 (5.0)	15 (6.6)
To GXR	6 (0.5)	—
To nonstimulant other than GXR	30 (2.7)	8 (3.5)
To a stimulant	44 (4.0)	14 (6.1)

^a^ Includes treatment discontinuation, augmentation, or switching.

AAP, atypical antipsychotic; GXR, guanfacine extended release.

**FIG. 3.**
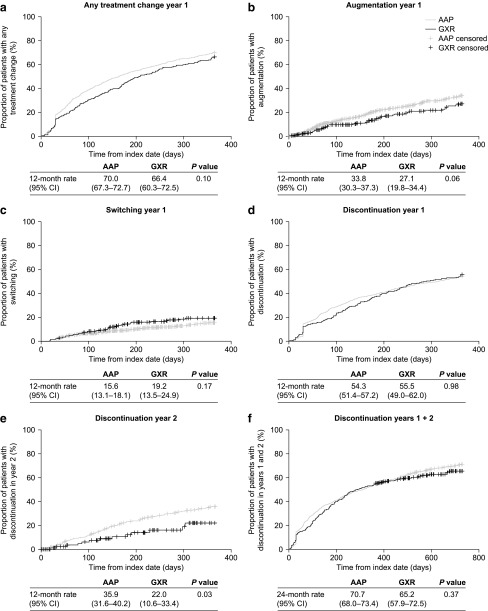
Kaplan–Meier rates of **(a)** any treatment change, **(b)** augmentation, **(c)** switching, **(d)** discontinuation during the first year after initiation of AAP or GXR, **(e)** discontinuation during the second year, and **(f)** discontinuation during the entire 2-year period. Number of patients included in each treatment group in year 1 and years 1 + 2 analyses: AAP, *n* = 1098; GXR, *n* = 229; year 2 analysis: AAP, *n* = 502; GXR, *n* = 102. *p-*value (AAP vs. GXR) based on log rank test. AAP, atypical antipsychotic; CI, confidence interval; GXR, guanfacine extended release.

In Kaplan–Meier analyses, 12-month rates for any of the treatment changes did not differ significantly between the AAP and GXR groups during the first year of follow-up (all *p* > 0.05) (Fig. 3a–d).

Discontinuation, which can be more frequent during long-term use of AAPs owing to chronic side effects (Rasimas and Liebelt 2012), was also analyzed during the second year of follow-up. The 24-month discontinuation rates over a 2-year follow-up were similar with AAPs and GXR (*p* > 0.05) (Fig. 3f). However, when assessed individually, the 12-month discontinuation rate over the second year of follow-up was significantly lower with GXR than with AAPs (22.0% vs. 35.9%; *p* = 0.03) (Fig. 3e).

### Health care resource utilization

Both in patients receiving AAPs and in those receiving GXR, the mean number of all-cause drug prescriptions per patient was significantly higher after initiation of the index treatment than before (AAP: 14.9 vs. 23.1; GXR: 13.7 vs. 19.5; all *p* < 0.01) (Table 3). Mean number of overall all-cause medical services used per patient was significantly higher in the 6 months after initiating an AAP than in the 6 months before (before, 4.0; after, 5.2; *p* < 0.01), whereas it remained similar in the GXR group (before, 4.0; after, 3.9; *p* = 0.24) (Table 3). The rise in all-cause medical services use among patients treated with an AAP was mainly a result of more psychiatric department visits after initiation of an AAP (1.5 vs. 2.6; *p* < 0.01).

**Table 3.** All-Cause Utilization of Health Care Resources in the 6 Months Before and 6 Months After Initiation of an Atypical Antipsychotic or Guanfacine Extended Release in Patients with ≥1 Service Use

*Number of services used per patient, mean (SD)*	*AAP (*n* = 1098)*			*GXR (*n* = 229)*		
	*6 Months before index treatment*	*6 Months after index treatment*	p^a^	*6 Months before index treatment*	*6 Months after index treatment*	p^a^
Inpatient admissions	0.1 (0.6)	0.2 (1.1)	0.23	0.1 (0.3)	<0.1 (0.2)	0.049
Inpatient days	0.4 (2.8)	0.8 (5.3)	0.46	0.1 (0.6)	0.1 (0.6)	0.20
Emergency department visits	0.4 (1.0)	0.4 (1.0)	0.11	0.2 (0.6)	0.2 (0.7)	0.92
Outpatient visits	1.9 (1.9)	1.9 (2.0)	0.92	2.5 (2.1)	2.3 (2.1)	0.10
Psychiatric department visits	1.5 (2.8)	2.6 (5.7)	<0.01	1.0 (2.3)	1.1 (3.9)	0.77
Other medical services^b^	0.1 (0.5)	0.1 (0.5)	0.11	0.2 (0.6)	0.2 (0.8)	0.76
All medical services	4.0 (3.7)	5.2 (6.1)	<0.01	4.0 (3.2)	3.9 (4.4)	0.24
Drug prescriptions	14.9 (20.6)	23.1 (24.7)	<0.01	13.7 (13.7)	19.5 (15.3)	<0.01

^a^ Health care resource utilizations were compared between the 6 months before and 6 months after initiation of the index treatment (Wilcoxon signed-rank tests).

^b^ Other medical services include services dispensed from a local community service center, a chronic pain center, a foster care establishment, or a laboratory.

AAP, atypical antipsychotic; GXR, guanfacine extended release; SD, standard deviation.

### Health care cost

With both AAPs and GXR, mean total health care cost per patient (all medical cost and prescription drug cost) was significantly higher in the 6 months after initiating the index treatment compared with the 6 months before (AAPs: before, C$1497.1; after, C$2288.3; *p* < 0.01; GXR: before, C$1378.6; after, C$1997.2; *p* < 0.01) (Table 4). Likewise, mean all-cause prescription drug cost per patient was significantly higher in the 6 months after initiation of either treatment than in the 6 months before, although the increase was numerically higher with GXR than with AAPs (AAPs: before, C$696.6; after, C$937.0; *p* < 0.01; GXR: before, C$761.1; after, C$1432.4; *p* < 0.01) (Table 4). However, mean overall medical cost per patient was significantly higher 6 months after initiating an AAP than 6 months before (before, C$800.5; after, C$1351.2; *p* < 0.01), but it decreased among patients using GXR (before, C$627.5; after, C$564.8; *p* = 0.07) (Table 4).

**Table 4.** All-Cause Health Care Cost in the 6 Months Before and 6 Months After Initiation of an Atypical Antipsychotic or Guanfacine Extended Release in Patients with ≥1 Service Use

*All-cause health care cost per patient, mean (SD)*	*AAP (*n* = 1098)*			*GXR (*n* = 229)*		
	*6 Months before index treatment*	*6 Months after index treatment*	p^a^	*6 Months before index treatment*	*6 Months after index treatment*	p^a^
Inpatient cost	480.0 (2927.9)	898.4 (5547.7)	0.61	138.4 (668.0)	100.2 (703.1)	0.18
Emergency department cost	107.0 (292.9)	96.5 (282.0)	0.18	60.1 (176.1)	62.8 (208.9)	0.96
Outpatient cost	147.3 (167.7)	157.8 (213.5)	0.30	298.8 (301.1)	254.7 (283.2)	0.02
Psychiatric department visit cost	167.2 (288.6)	287.8 (575.5)	<0.01	170.1 (377.0)	194.2 (690.9)	0.65
Other medical cost^b^	6.1 (36.9)	7.3 (43.2)	0.44	10.3 (50.2)	15.6 (121.1)	0.92
Overall medical cost	800.5 (2982.8)	1351.2 (5658.7)	<0.01	627.5 (885.0)	564.8 (1049.1)	0.07
Prescription drug cost	696.6 (653.2)	937.0 (710.3)	<0.01	761.1 (359.4)	1432.4 (495.1)	<0.01
Total health care cost	1497.1 (3065.8)	2288.3 (5691.8)	<0.01	1378.6 (968.4)	1997.2 (1168.4)	<0.01

All costs are in Canadian dollars.

^a^ Health care costs were compared between the 6 months before and 6 months after initiation of the index treatment (Wilcoxon signed-rank tests).

^b^ Other medical cost includes costs dispensed from a local community service center, a chronic pain center, a foster care establishment, or a laboratory.

AAP, atypical antipsychotic; GXR, guanfacine extended release; SD, standard deviation.

The increase in overall medical cost among AAP users was driven mainly by significantly higher costs associated with psychiatric department visits (before, C$167.2; after C$287.8; *p* < 0.01).

### Subanalysis in patients with index date December 1, 2013, or later

To assess treatment changes, HCRU, and health care cost in the AAP and GXR groups over the same time period, a subanalysis was performed in patients with an index date December 1, 2013 (i.e., the earliest index date in the GXR group), or later (Supplementary Table S3). Compared with the full data set, the distribution of patient characteristics across the AAP and GXR groups remained similar in the post-December subpopulation, except for a lower proportion of recipients of last-resort financial assistance in the AAP subgroup (37.0%; overall population, 42.2%).

Rates of index treatment changes with AAPs and GXR observed in the post-December 2013 subpopulation were similar to those observed in the overall population (Supplementary Table S4), but GXR was more frequently included in secondary treatment changes in the AAP subgroup (∼5%; overall population <1%), reflecting market uptake of GXR.

Results for changes in HCRU and health care cost were also consistent between the post-December 2013 subgroup and the full data set (Supplementary Tables S5 and S6).

### Factors associated with a change in health care cost after initiation of the index treatment

In a linear regression analysis, initiation of GXR was associated with a significantly greater 6-month rise in prescription drug cost than AAPs (adjusted difference, C$516; *p* < 0.01), but there was no significant difference between the two treatments for total health care cost (AAP vs. GXR adjusted difference, C$−64; *p* = 0.87) (Supplementary Table S7). The only factor significantly associated with a change in total health care cost 6 months after initiation of the index treatment compared with 6 months before was a high number of comorbidities (0 vs. ≥3 comorbidities adjusted difference, C$3845; *p* < 0.01).

### Trends in health care cost 2 years before and 2 years after the introduction of GXR in Canada

Overall trends in total health care cost did not differ significantly between the 2-year periods before and after introduction of GXR in the Quebec formulary (Supplementary Fig. S1). Before GXR introduction, mean total health care costs increased by C$1.1 per 3 months, compared with C$5.3 per 3 months after the introduction of GXR (rate difference: C$4.2, *p* = 0.30).

## Discussion

The present study uses claims data from a large, governmental health care plan to compare the impact on treatment patterns, HCRU, and health care costs associated with switching stimulants to, or augmenting stimulants with, AAPs or GXR for the management of ADHD in children and adolescents in clinical practice in Quebec. Secondary treatment changes were similar for both treatments after 1 year, but discontinuation was significantly lower in patients using GXR than in those using AAPs during the second year. Although prescription costs were higher for GXR than AAPs, there was no overall difference in total health care cost between the two treatments.

The antecedent RAMQ database analysis of data from 2007 to 2012 found that HCRU and health care cost increased significantly in children and adolescents with ADHD who initiated AAPs after stimulant therapy (Lachaine et al. 2014). AAPs are the most common psychotropic medication used to augment a stimulant and the second most common medication when switching from a stimulant (Ben Amor et al. 2014). GXR is an approved medication for ADHD, which has been available on the Canadian formulary since February 2014; drug utilization experience for GXR in Canada is therefore limited.

In both the AAP and GXR groups, secondary treatment changes of the index treatment were frequent, with about 70% of patients modifying their regimen during the first year. The probability of treatment discontinuation, the most common treatment change during year 1, was similar with AAP and GXR over a 2-year follow-up (24-month rates ∼65%–70% in each group). However, when the two follow-up years were assessed separately, the estimated 12-month discontinuation rate during year 2 was significantly lower with GXR (22.0%) than with AAPs (35.9%).

The higher discontinuation rate with AAPs compared with GXR in the second year of treatment may be due to long-term sequelae of side effects, in particular metabolic complications, and a waning efficiency during prolonged AAP use (CADDRA 2011). Indeed, although in the short term AAPs may reduce aggression and disruptive behavior in children and adolescents with ADHD (Aman et al. 2004; Armenteros et al. 2007; Loy et al. 2017), there is little evidence supporting the long-term effectiveness and safety of AAPs in pediatric populations with ADHD (Pringsheim et al. 2011c). On the other hand, increases in weight and BMI have been consistently demonstrated in pediatric patients receiving AAPs, and have been shown to persist during long-term therapy (Pringsheim et al. 2011c; Rasimas and Liebelt 2012).

Despite generic AAPs having been available for most of the duration of this study, initiation of GXR did not result in a significantly greater increase in total health care cost than the use of AAPs. GXR use was associated with a greater increase in prescription drug cost over the 6-month follow-up than use of AAPs, but this was balanced by the greater increase in overall medical cost with AAPs (mainly attributed to more psychiatric department visits), resulting in a similar increase in total health care cost with both treatments. Consistent with this finding, the overall trend in total health care cost in Quebec did not increase significantly in the 2 years after introduction of GXR in Canada compared with the 2 years before. Considering that the difference in discontinuation rates between GXR and AAPs did not become evident until the second treatment year, a longer follow-up may result in a greater effect of GXR use on cost outcomes.

Two other studies conducted in the USA also compared treatment patterns and cost between AAPs and other medications for management of ADHD. A claims database study from 2005 to 2009 assessed children (6–12 years of age) with ADHD who had changed their stimulant regimen to include an AAP or a non-AAP (mostly ATX or clonidine). Over a 1-year follow-up, this study reported significantly lower mean total health care cost (including lower mean medical cost and mean prescription drug cost) with non-AAPs compared with AAPs (Sikirica et al. 2012b). In the present study, total health care costs were not different between AAPs and GXR after 6 months, but, as discussed above, over a longer follow-up, a greater economic effect of GXR (i.e., a reduction in total cost) may become evident. In addition, the U.S. study reported higher 1-year rates of switching and augmentation with AAPs (17.2% and 43.4%, respectively) than non-AAPs (10.4% and 22.4%, respectively) (Sikirica et al. 2012b), whereas in the present study, they were similar with AAPs (15.6% and 33.8%, respectively) and GXR (19.2% and 27.1%, respectively).

The different treatment patterns in the two studies could be due to inclusion of an older patient population (6–17 years) in the present study, possibly associated with a higher proportion of psychotic comorbid diagnoses, and the different type of non-AAP used, which may have impacted on the likelihood of the regimen being modified. In addition, propensity score matching of the treatment groups in the U.S. study may have eliminated confounding factors, and differences in treatment patterns and costs may have become apparent sooner.

In another U.S. study comparing the cost-effectiveness of AAPs, clonidine ER or GXR, and ATX in children and adolescents with ADHD who had failed stimulant treatment, clonidine ER or GXR was the most cost-effective strategy (C$4997.89/quality-adjusted life-years [QALY]), whereas AAPs were the least cost-effective (C$8254.76/QALY); ATX was associated with a cost of C$5051.06/QALY (Sohn et al. 2016b).

A number of limitations should be considered when interpreting the present study data. First, the difference between the study start date (January 2007) and the earliest GXR index date (December 2013) resulted in a larger sample size and longer follow-up of the AAP group than the GXR group. Between 2007 and 2013 (i.e., the period covered by the AAP group only), several formulary changes occurred (e.g., generic versions of AAPs became available), which could have affected treatment patterns and HCRU among AAP users. However, AAP and GXR users were generally well balanced in key demographic and clinical characteristics, such as gender, age, and comorbidities. The post-December 2013 subgroup analysis was included to address disparities between the different cohorts, with balanced sample sizes between the two treatment groups. In the subgroup analysis, HCRU and cost outcomes were similar to those in the overall study population.

A second limitation is that there were more recipients of financial assistance among AAP users than among GXR users. This could suggest that a lower socioeconomic background might have impacted on the likelihood of receiving generic medications (i.e., AAPs), and possibly also on the likelihood of receiving health care services. However, prescription claims data were derived from the public prescription drug plan (i.e., patients with private health care were not included in the study); therefore, both index treatment groups had the same medication coverage. Importantly, the post-December 2013 subgroup analysis, in which proportions of financial assistance recipients were similar in the two treatment groups, confirmed the HCRU and cost findings observed with the full data set. In addition, the regression analysis showed that receipt of financial assistance was not associated with a significant change in health care cost.

Third, because ADHD severity is not captured in the RAMQ database, the possibility that the AAP group may have included a higher proportion of difficult-to-treat patients, and hence incurred higher costs, than the GXR group cannot be excluded. Indeed, there was a difference in the distribution of the prescribing physicians' specialties between the two treatment groups, with GXR generally prescribed by pediatricians, and AAPs prescribed by psychiatrists or pediatricians, which could be indicative of a more complex pathology. However, in the regression analysis, having the index treatment prescribed by a specialist (psychiatrist, pediatrician, or neurologist) was not associated with a significant rise in total health care cost compared with receiving the prescription from a general practitioner. The only factor associated with a significant cost increase was the mean number of comorbidities, which was similar across treatment groups.

Finally, there are several limitations associated with the use of a claims database. For example, the use of an indirect measure of treatment patterns based on claims data may not necessarily correspond to the actual drug consumption by the patient. Furthermore, medications taken by patients outside of the RAMQ drug formulary, as well as nonmedical interventions that might have affected patients' outcomes, were not accounted for in these analyses.

## Conclusion

In children and adolescents with ADHD whose stimulant treatment was modified by initiating AAPs or GXR, rates of secondary treatment changes were similar in both groups over the first year of follow-up, but discontinuation was significantly less frequent with GXR than with AAPs in the second year. Despite a greater increase in prescription cost with GXR, there was overall no difference in total health care cost between the two treatments, because patients receiving AAPs used more medical services and incurred higher medical costs. The findings may help to improve overall patient outcomes and optimize HCRU in the management of ADHD.

## Clinical Significance

Overall health care costs and HCRU were similar for GXR compared with AAPs in children and adolescents with ADHD whose treatment was modified by switching or augmentation. Significantly less discontinuation was observed in patients receiving GXR compared with AAPs. These findings may help to improve management of ADHD.

## Data Access

The data included in this study were purchased from the RAMQ and are not publicly available. Data analyses are available from Shire LLC, a Takeda company, upon request.

## Supplementary Material

Supplemental data

Supplemental data

Supplemental data

Supplemental data
