# Supplementary material for: Treatment Patterns, Health Care Resource Utilization, and Health Care Cost Associated with Atypical Antipsychotics or Guanfacine Extended Release in Children and Adolescents with Attention-Deficit/Hyperactivity Disorder in Quebec, Canada
Source: J Child Adolesc Psychopharmacol. 2019 Dec 2;29(10):730–9. doi: 10.1089/cap.2019.0097 (PMC6885769; doi:10.1089/cap.2019.0097)
Supplement: Supplemental data [file Supp_Fig1-TableS1.pdf]

Supplementary Data

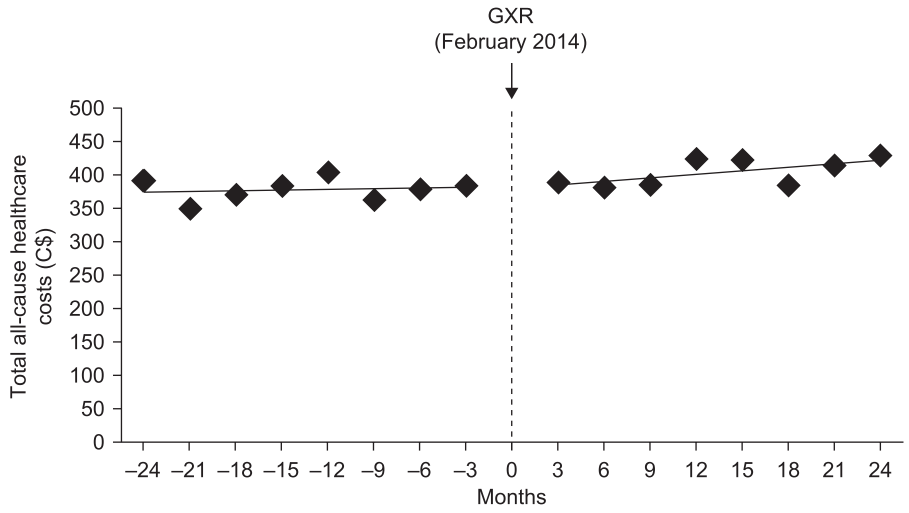

SUPPLEMENTARY FIG. S1. Trends in health care cost from February 2012 to February 2016. Mean total all-cause health care costs per 3 months (in Canadian dollars) are shown. The arrow represents the introduction of GXR in the Quebec formulary. AAP, atypical antipsychotic; GXR, guanfacine extended release.

Supplementary Table S1. Types of Index Stimulants

Index stimulant[a]	*AAP* (n = *1098*)	*GXR* (n = *229*)
Amphetamine, *n* (%)		
Amphetamine instant release		
Dexamphetamine (Dexedrine)	24 (2.2)	6 (2.6)
Amphetamine extended release		
Amphetamine (Adderall XR)	186 (16.9)	20 (8.7)
Dexamphetamine (Dexedrine)	40 (3.6)	1 (0.4)
Lisdexamfetamine (Vyvanse)	128 (11.7)	97 (42.4)
Methylphenidate, *n* (%)		
Methylphenidate instant release		
Methylphenidate (generic)	291 (26.5)	20 (8.7)
Methylphenidate (Ritalin)	18 (1.6)	0 (0)
Methylphenidate extended release		
Methylphenidate (generic)	36 (3.3)	3 (1.3)
Methylphenidate (Biphentin)	92 (8.4)	37 (16.2)
Methylphenidate (Concerta)	272 (24.8)	45 (19.7)
Methylphenidate (Ritalin SR)	11 (1.0)	0 (0)
Instant release drugs, *n* (%)	333 (30.3)	26 (11.4)
Extended release drugs, *n* (%)	765 (69.7)	203 (88.6)
Number of distinct stimulants, mean (SD) [median]	1.5 (0.7) [1.0]	1.6 (0.7) [1.0]

[a]Defined as the stimulant used before initiation of the index treatment (AAP or GXR).

AAP, atypical antipsychotic; GXR, guanfacine extended release; SD, standard deviation; SR, sustained release; XR, extended release.
