# Supplementary material for: Treatment Patterns, Health Care Resource Utilization, and Health Care Cost Associated with Atypical Antipsychotics or Guanfacine Extended Release in Children and Adolescents with Attention-Deficit/Hyperactivity Disorder in Quebec, Canada
Source: J Child Adolesc Psychopharmacol. 2019 Dec 2;29(10):730–9. doi: 10.1089/cap.2019.0097 (PMC6885769; doi:10.1089/cap.2019.0097)
Supplement: Supplemental data [file Supp_TableS2-S3.pdf]

Supplementary Table S2. Physician's Specialty

Physician's specialty, n *(%)*	*AAP* (n=*1098)*	*GXR* (n=*229)*
Physician prescribing index treatment		
Psychiatrist	346 (31.5)	32 (14.0)
Pediatrician	270 (24.6)	116 (50.7)
Neurologist	41 (3.7)	1 (0.4)
General Practitioner	165 (15.0)	32 (14.0)
Others	273 (24.9)	48 (21.0)
Missing	3 (0.3)	0 (0.0)
Physician prescribing ≥1 stimulant during baseline period[a]		
Psychiatrist	368 (33.5)	46 (20.1)
Pediatrician	548 (49.9)	158 (69.0)
Neurologist	47 (4.3)	1 (0.4)
General Practitioner	369 (33.6)	57 (24.9)
Physician providing ≥1 ADHD diagnosis during baseline period[a]		
Psychiatrist	327 (29.8)	41 (17.9)
Pediatrician	380 (34.6)	120 (52.4)
Neurologist	40 (3.6)	1 (0.4)
General Practitioner	192 (17.5)	41 (17.9)

Only the most common physician specialties are reported.

[a]Categories are not mutually exclusive, as patients could have been seen by more than one specialist during the baseline period.

AAP, atypical antipsychotic; ADHD, attention-deficit/hyperactivity disorder; GXR, guanfacine extended release.

Supplementary Table S3. Demographics and Patient Characteristics at Index Date Among Patients with Index Date December 1, 2013 or Later

	AAP (n=*154)*	*GXR* (n=*229)*
Sociodemographics		
Age, years, mean (SD)	10.7 (3.0)	10.1 (2.2)
Age groups, years, *n* (%)		
6–12	103 (66.9)	185 (80.8)
13–17	51 (33.1)	44 (19.2)
Male, *n* (%)	116 (75.3)	175 (76.4)
Recipients of last-resort financial assistance,[a] *n* (%)	57 (37.0)	59 (25.8)
Number of comorbidities, *n* (%)		
0	72 (46.8)	124 (54.1)
1	62 (40.3)	79 (34.5)
2	17 (11.0)	20 (8.7)
≥3	3 (1.9)	6 (2.6)
Comorbidity profile, *n* (%)		
Adjustment disorder	15 (9.7)	9 (3.9)
Anxiety disorder	8 (5.2)	9 (3.9)
Conduct disorder	4 (2.6)	12 (5.2)
Depression	6 (3.9)	2 (0.9)
Insomnia	2 (1.3)	4 (1.7)
Learning disability	3 (1.9)	11 (4.8)
Obsessive–compulsive disorder	0 (0)	1 (0.4)
Oppositional defiant disorder	4 (2.6)	0 (0)
Pervasive developmental disorders	2 (1.3)	4 (1.7)
Tics	2 (1.3)	10 (4.4)
Substance abuse	2 (1.3)	2 (0.9)
Epilepsy	0 (0)	1 (0.4)
Other neurological disorders	0 (0)	3 (1.3)
Accidents and injuries	54 (35.1)	66 (28.8)
Asthma	3 (1.9)	3 (1.3)

[a]Beneficiaries of the social assistance program.

AAP, atypical antipsychotic; GXR, guanfacine extended release; SD, standard deviation.
