# Supplementary material for: Treatment Patterns, Health Care Resource Utilization, and Health Care Cost Associated with Atypical Antipsychotics or Guanfacine Extended Release in Children and Adolescents with Attention-Deficit/Hyperactivity Disorder in Quebec, Canada
Source: J Child Adolesc Psychopharmacol. 2019 Dec 2;29(10):730–9. doi: 10.1089/cap.2019.0097 (PMC6885769; doi:10.1089/cap.2019.0097)
Supplement: Supplemental data [file Supp_TableS4-S5.pdf]

Supplementary Table S4. Changes of Index Treatment During the 12-Month Follow-Up Among Patients with Index Date December 1, 2013, or Later

Type of change, n *(%)*	*AAP* (n = *154)*	*GXR* (n = *229)*
Any change in index treatment[a]	115 (74.7)	152 (66.4)
Index treatment discontinuation	93 (60.4)	127 (55.5)
Index treatment augmentation	33 (21.4)	42 (18.3)
With an AAP	2 (1.3)	8 (3.5)
With GXR	3 (1.9)	—
With a nonstimulant other than GXR	3 (1.9)	2 (0.9)
With a stimulant	25 (16.2)	32 (14.0)
Index treatment switching	24 (15.6)	37 (16.2)
To an AAP	7 (4.5)	15 (6.6)
To GXR	5 (3.2)	—
To nonstimulant other than GXR	4 (2.6)	8 (3.5)
To a stimulant	8 (5.2)	14 (6.1)

[a]Included treatment discontinuation, augmentation, or switching.
AAP, atypical antipsychotic; GXR, guanfacine extended release.

Supplementary Table S5. All-Cause Utilization of Health Care Resources in the 6 Months Before and 6 Months After Initiation of an Atypical Antipsychotic or Guanfacine Extended Release Among Patients with Index Date December 1, 2013, or Later

	AAP (n = *154)*			*GXR* (n = *229)*		
Number of services used per patient, mean (SD)	*6 Months before index treatment*	*6 Months after index treatment*	p[a]	*6 Months before index treatment*	*6 Months after index treatment*	p[a]
Inpatient admissions	0.1 (0.4)	0.1 (0.4)	0.81	0.1 (0.3)	<0.1 (0.2)	0.049
Inpatient days	0.1 (0.5)	0.5 (3.5)	0.84	0.1 (0.6)	0.1 (0.6)	0.20
Emergency department visits	0.3 (0.7)	0.3 (0.8)	0.37	0.2 (0.6)	0.2 (0.7)	0.92
Outpatient visits	1.7 (1.7)	1.9 (2.3)	0.36	2.5 (2.1)	2.3 (2.1)	0.10
Psychiatric department visits	1.4 (2.7)	2.7 (4.8)	<0.01	1.0 (2.3)	1.1 (3.9)	0.77
Other medical services[b]	0.2 (0.6)	0.2 (0.6)	0.53	0.2 (0.6)	0.2 (0.8)	0.76
All medical services	3.7 (3.4)	5.1 (5.3)	<0.01	4.0 (3.2)	3.9 (4.4)	0.24
Prescription drugs	16.5 (20.1)	26.9 (27.9)	<0.01	13.7 (13.7)	19.5 (15.3)	<0.01

[a]Health care resource utilizations were compared between the 6 months before and 6 months after initiation of the index treatment (Wilcoxon signed-rank tests).
[b]Other medical services include services dispensed from a local community service center, a chronic pain center, a foster care establishment, or a laboratory.
AAP, atypical antipsychotic; GXR, guanfacine extended release; SD, standard deviation.
