# Supplementary material for: Treatment Patterns, Health Care Resource Utilization, and Health Care Cost Associated with Atypical Antipsychotics or Guanfacine Extended Release in Children and Adolescents with Attention-Deficit/Hyperactivity Disorder in Quebec, Canada
Source: J Child Adolesc Psychopharmacol. 2019 Dec 2;29(10):730–9. doi: 10.1089/cap.2019.0097 (PMC6885769; doi:10.1089/cap.2019.0097)
Supplement: Supplemental data [file Supp_TableS6-S7.pdf]

Supplementary Table S6. All-Cause Health Care Cost in the 6 Months Before and 6 Months After Initiation of an Atypical Antipsychotic or Guanfacine Extended Release in Patients with ≥1 Services Use Among Patients with Index Date December 1, 2013 or Later

	AAP (n = *154*)			*GXR* (n = *229*)		
All-cause health care cost per patient, mean (SD)	*6 Months before index treatment*	*6 Months after index treatment*	p[a]	*6 Months before index treatment*	*6 Months after index treatment*	p[a]
Inpatient cost	166.2 (634.4)	593.1 (3731.8)	0.97	138.4 (668.0)	100.2 (703.1)	0.18
Emergency department cost	98.0 (226.2)	91.5 (252.3)	0.53	60.1 (176.1)	62.8 (208.9)	0.96
Outpatient cost	167.0 (200.1)	203.3 (318.8)	0.21	298.8 (301.1)	254.7 (283.2)	0.02
Psychiatric department visit cost	246.4 (415.3)	379.6 (598.3)	0.01	170.1 (377.0)	194.2 (690.9)	0.65
Other medical cost[b]	12.1 (60.8)	14.6 (86.2)	0.76	10.3 (50.2)	15.6 (121.1)	0.92
All medical cost	591.6 (852.5)	1190.7 (4003.1)	0.03	627.5 (885.0)	564.8 (1049.1)	0.07
Prescription drug cost	704.4 (500.3)	977.9 (560.6)	<0.01	761.1 (359.4)	1,432.4 (495.1)	<0.01
Total health care cost	1296.0 (1079.0)	2168.6 (4116.2)	<0.01	1378.6 (968.4)	1997.2 (1168.4)	<0.01

All costs are in Canadian dollars.
[a]Health care costs were compared between the 6 months before and 6 months after initiation of the index treatment (Wilcoxon signed-rank tests).
[b]Other medical cost includes costs dispensed from a local community service center, a chronic pain center, a foster care establishment, or a laboratory.
AAP, atypical antipsychotic; GXR, guanfacine extended release; SD, standard deviation.

Supplementary Table S7. Linear Regression Analysis of Factors Associated with a Change in Cost 6 Months After Initiation of the Index Medication Compared with the 6 Months Before

	Adjusted difference in cost (p)		
Variables	*All medical cost*	*Prescription drug cost*	*Total health care cost*
Type of index treatment			
AAP	Reference	Reference	Reference
GXR	−579 (0.13)	516 (<0.01)	−64 (0.87)
Class of physician prescribing the index treatment			
General practitioner	Reference	Reference	Reference
Psychiatrist	461 (0.18)	85 (0.06)	546 (0.12)
Pediatrician	−465 (0.18)	28 (0.55)	−437 (0.21)
Neurologist	−891 (0.28)	−129 (0.23)	−1019 (0.22)
Age (1 year difference)	37 (0.46)	44 (<0.01)	81 (0.11)
Sex (male vs. female)	525 (0.12)	25 (0.57)	550 (0.11)
Last-resort financial assistance	−386 (0.19)	−51 (0.19)	−437 (0.14)
Number of comorbidities			
0	Reference	Reference	Reference
1	377 (0.23)	78 (0.06)	455 (0.15)
2	1084 (0.02)	−20 (0.75)	1064 (0.02)
≥3	3738 (<0.01)	107 (0.28)	3845 (<0.01)

AAP, atypical antipsychotic; GXR, guanfacine extended release.
